# Total hip arthroplasty for dysplastic coxarthrosis using a cementless Wagner Cone stem

**DOI:** 10.1186/s10195-021-00578-8

**Published:** 2021-04-17

**Authors:** Giuseppe Solarino, Giovanni Vicenti, Andrea Piazzolla, Francesco Maruccia, Angela Notarnicola, Biagio Moretti

**Affiliations:** Department of Basic Medical Sciences, Neuroscience and Organs of Sense, School of Medicine, AOU Policlinico Consorziale, Università Di Bari “AldoMoro”, Piazza Giulio Cesare n.11, 70124 Bari, Italy

**Keywords:** Hip, Dysplasia, Total hip arthroplasty, Conical stem

## Abstract

**Background:**

Total hip arthroplasty (THA) is currently the best surgical option for hip osteoarthritis secondary to developmental hip dysplasia (DDH); it may be extremely challenging, because of the hypoplasic proximal metaphysis, pathological anteversion, and excessive cervico-diaphyseal angle of the neck at the femoral side. The purpose of this retrospective study was to evaluate the long-term survival and clinical and radiological results of Conus uncemented stems, implanted in patients affected by hip osteoarthritis with Crowe not-type IV secondary to DDH.

**Material and methods:**

We identified 100 consecutive THAs performed for DDH in 63 women and 24 men, with an average age of 53 years in a single center. Thirteen patients underwent bilateral hip replacement. The patients’ mean body mass index was 29.8 kg/m^2^ (range 27.1–35.6 kg/m^2^). The main indications for surgery were severe hip pain and considerable functional impairment: the preoperative Harris Hip Score was 29.5 on average (range 22–61). Radiologically, 8 hips were classified as Crowe I, 43 hips as Crowe II, and 49 hips as Crowe III. In all cases, we implanted the Wagner femoral cone prosthesis using the direct lateral approach; in the attempt to reestablish native hip biomechanics, 66 stems were 135° and 34 were 125°.

**Results:**

The mean follow-up of the study was 11.7 years (range 2.2–21.8 years). Harris Hip Score increased to a mean value of 71.5 points (range 52–93 points). Radiographic evaluation demonstrated osteointegration of the implant with stable bone growth observed at the stem–endosteum interface; signs of bone readaptation and thinning of the femoral calcar were present in nine hips. None of the patients underwent revision for septic or aseptic loosening of the stem; none sustained a periprosthetic fracture.

**Conclusions:**

This study confirms the theoretical advantages that suggest the choice of the Wagner cone when technical difficulties during prosthetic surgery are expected owing to abnormal proximal femoral anatomy.

**Level of evidence:**

Level IV, retrospective case study

## Introduction

Total hip arthroplasty (THA) is currently the best surgical option for hip osteoarthritis due to longstanding developmental hip dysplasia (DDH); during surgery, certain problems may be extremely challenging, such as poor bone stock at the shallow and roofless acetabular side and at the hypoplasic proximal metaphysis, with larger anteroposterior diameter compared with the mediolateral and pathological anteversion, and excessive cervico-diaphyseal angle of the neck at the femoral side. Besides osseous deformities, surrounding soft tissues may exhibit scarring and fibrosis with damaged or severely contracted muscles [1].

Furthermore, pelvic and/or proximal femoral osteotomies might have been performed previously in pediatric patient populations in the attempt to correct structural deformities and to improve the mechanics of the hip, and consequently decrease pain and achieve better function [2, 3]. Surgeons should take note of previous scars and must pay attention to the location of the hardware that might have been left in situ and be prepared to remove it, if required, keeping in mind that a different approach to the hip, such as the conventional, may be more advisable. Therefore, primary hip arthroplasty in sequelae of DDH requires elaborate preoperative planning, must be considered technically demanding, and, if the patients require leg lengthening of more than 4 cm, shortening osteotomy of the femur is recommended, and particularly challenging in terms of implant survival [4–6].

On the femoral side, the use of a stem with a rounded section, whose design consists of a proximally conical and tapered prosthesis for better load distribution with the sharp longitudinal ribs and the Titanium alloy coarse-blasted surface to enhance stability and osseointegration, should allow immediate fixation in the medullary bony bed and restoration of the proper version angle during insertion, thanks to its narrow round proximal cross-section that facilitates adjustment of the anteversion angle of the stem, especially in patients whose femoral geometry precludes the use of standard-sized implants and for whom metaphyseal engaging stems are not an ideal choice because of DDH [7–9]. Finally, being a monoblock, it excludes the theoretical disadvantage of potential for metal corrosion given by modular-designed components that also may be recommended to accommodate the shape of the dysplastic canal [2, 10, 11].

The purpose of this retrospective study was to evaluate the long-term survival and clinical and radiological results of 100 Conus uncemented stems, implanted in patients affected by hip osteoarthritis with Crowe not-type IV secondary to DDH.

## Materials and methods

We identified 100 consecutive THAs performed for DDH in a single center where the Wagner Cone stem was used; For each patient, complete medical history was collected. The main indications for surgery were severe hip pain and considerable functional impairment, while restoration of leg length discrepancy was not considered as a primary goal. Exclusion criteria included patients with less than 2-year minimum follow-up, patients with active or previous hip joint infection, and patients with a history of neuromuscular disorder who could not participate in standard rehabilitation protocols. This study was approved by the institutional review board at our university. The approval of the ethics committee had not been requested, in consideration of the retrospective study design. Study protocol was in accordance with the Declaration of Helsinki for human research.

The majority of the patients were female, that is, 63 women and 24 men with a mean age of 53 years (range 27–88 years); all of them were identified as white/Caucasian. Thirteen patients underwent bilateral THA consecutively, not simultaneously. The right hip was operated in 64 cases, the left in 36. The patients’ mean body mass index (BMI) was 29.8 (range 27.1–35.6).

Informed consent was obtained from all patients. Preoperative clinical assessment was done using the Harris Hip Score (HHS); the preoperative HHS was 29.5 on average (range 22–61). For radiological evaluation, anteroposterior pelvic radiographs and axial view of the involved hip were obtained to evaluate the acetabular bone stock, and to estimate the predicted cup coverage and size. Templating was performed by two fellowship-trained orthopedic surgeons on their respective patients to select the appropriately sized implant. The teardrop on the deformed side was considered as the more convenient marker of the acetabular cup desirable position. When necessary, computed tomography (CT) images were used for further investigation, mainly to determine the thickness of the medial wall and of the elusive anterior wall. Although two main classification systems have been proposed to describe the severity of DDH in adults, because they seem to demonstrate good interobserver and intraobserver agreement, thus suggesting the use of both to increase the preoperative accuracy [12], we based dysplasia evaluation on the Crowe classification [13]: 8 hips were classified as Crowe I, 43 hips as Crowe II, 49 hips as Crowe III, and no hips as Crowe IV.

All patients were administered an antibiotic prophylaxis intravenously using 2 g of cefazolin during the operation and 1 g every 6 h for a total of three postoperative doses. In general, anesthesia or combined spinal-epidural anesthetic was administered—varying across patients depending on the anesthesiologist attending, patient’s preoperative comorbidities, and concomitant spinal pathology. All the THAs were implanted using the direct lateral approach (with patient laying in lateral decubitus position on the contralateral unaffected side) in a conventional turbulent flow theater. In total, 61 procedures were performed by the two former chiefs of the unit, while 39 cases were done by four surgeons among the senior registrars, experienced in joint replacement surgery; none of the THAs was performed by a trainee as first surgeon, even under supervision. The primitive acetabulum was reamed in or close to the anatomical position, and the acetabular cup was press-fit fixed with an intended inclination angle of 40–50° and an intended anteversion of 10–20°. We implanted only cementless hemispherical cups; the Zimmer-Biomet Continuum shell was the most common acetabular component, used 27 in cases, a Zimmer-Biomet Trilogy shell was used in 22 cases, and a Zimmer-Biomet TMT modular shell was used in 18 cases. Other acetabular components used included Protek Allocor in 13 cases, Protek Fitek in 10, Sulzer Fitmore in 4, Link Top in 3, Aesculap Plasmacup in 2, and Centrepulse Allofit in 1. Two or more additional acetabular screws were used in 71 cases to implement fixation based on the bone quality of the patient and the preference of the surgeon, but without any clear correlation with preoperative severity of Crowe classification. None of the cups required superolateral bone autograft augmentation to increase acetabular coverage. Table 1 summarizes the diameters of the cup implanted, the sizes of the inserted femoral cone stem, the diameters of the head used, and the coupling bearings chosen.

**Fig. 1:**
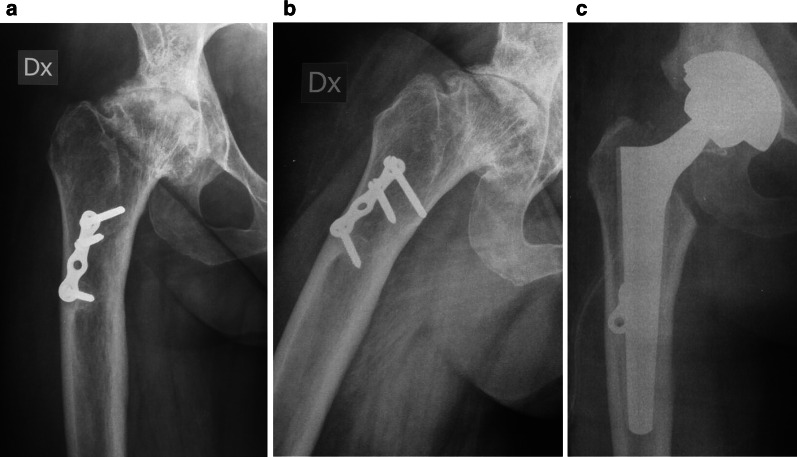
**a**, **b**, Preoperative AP view of the hip and lateral view of the femur in a coxarthrosis classified as Crowe I; a femoral proximal osteotomy had been previously performed in another country. **c**, Postoperative AP view of the operated hip; hardware were partially left in place

On the femoral side, in all cases we implanted the Wagner femoral cone prosthesis (Zimmer-Biomet, Warsaw, IN, USA); it is a short, diaphyseal engaging femoral stem, with titanium alloy coarse-blasted surface, with a 5° taper, available in two different neck angles (125° and 135°) and with lengths from 100.5 mm to 110 mm and in diameters from 6.4 mm to 10.4 mm (distal section distance: at 96 mm distance from the shoulder of the prosthesis) [14, 15]. In our case series, 66 THAs were 135° and 34 were 125° in the attempt to reestablish native hip biomechanics. In 11 cases, hardware from a previous femoral osteotomy were still in situ and were therefore removed if necessary (Fig. 1). A single tray of reamers and limited instrumentation affords a streamlined process for ease of use and turnover in the operating room. Progressive conical reamers were used manually to appropriately broach the femoral medullary canal until resistance against the inner cortex was felt; flexible reamers were never used to open up the canal prior to using them. Because restoration of anteversion is difficult to plan preoperatively, surgeon was guided by intraoperative trial; a combined anteversion that is less than 55° is an effective way to avoid dislocation after surgery [1]. Thus, the uncemented stem was inserted by guiding it with its appropriate device, aiming to obtain approximately 10–20° of anteversion. The prosthesis was rotated into the desired anteversion and was impacted into its definitive position. Then, the final femoral head was assembled manually and fixed to the taper of the femoral component with adequate hammer blows. Before closure in layers above one intraarticular drain, the stability of the hip implant was assessed using the shuck test and examining the primary arc range of motion. Perioperative care was the same for all patients: thromboembolic prophylaxis with low-molecular-weight heparin was administered for 5 weeks; during this period, patients used compression stockings. No medications were given to prevent heterotopic ossifications. Passive motion exercises with the assistance of a therapist started immediately after the operation, and the single intraarticular suction drain was removed on the second postoperative day. Patients were free to walk with two supports after 3 days for about 6 weeks, and thereafter, full weight-bearing was usually allowed.

Radiologically, any sign subsidence or tilting/migration of the femoral component was documented. AP pelvis and axial view of the involved hip were obtained and assessed by the same observer, who had not been involved in any treatment of patients enrolled in the study. The parameters investigated included alignment of the stem, subsidence of the stem, calcar resorption, and progression of radiolucent lines. Signs of stem subsidence or tilting/migration around femoral component were documented; our cutoff defining vertical migration was set at 3 mm, as previously reported [16]. Radiolucent lines were recorded using Gruen’s zones [17]; assessment of the mechanical implant stability completed the investigation of signs of stress-shielding and osseointegration, as supported by Engh [18]. Heterotopic ossifications were classified according to Brooker et al. [19]. The orientation of the component was classified as valgus, slight valgus, neutral, slight varus, or varus. Slight varus or slight valgus alignment was used to describe a femoral stem with less than 5° of malalignment with respect to the neutral axis of the femoral canal [20].

All eventual local complication, such as periprosthetic infection, dislocation, intra- and postoperative periprosthetic fracture, and breakages of the liner and/or of the head, were recorded. A Kaplan–Meier cumulative survival curve was processed to describe the probability of survival for the Wagner cone femoral stem prosthesis. It was generated by GraphPad Prism 9 Software (GraphPad Software, San Diego, CA, USA) and adopted to evaluate the survival of the implant both for aseptic loosening and for any cause in patients observed from the time of the surgical procedure until the end of follow-up. A *P*-value < 0.05 was considered significant.

## Results

All patients completed the HHS at a minimum of 2 years postoperation. The mean follow-up of the study was 11.7 years (range 2.2–21.8 years). Nine patients with 11 THAs died or were lost at the final follow-up at the study census date (January 2020); the two patients with bilateral THA were lost at 11 and 15 years of follow-up; the remaining seven patients with unilateral THA not eligible were lost after 5, 8 (two patients), 9, 12, 14, and 15 years of follow-up. Therefore, for the present study, 89 THAs were eligible in 91 patients; among these, all of them declared that they were satisfied with the results of surgery, showed clinical improvement, and walked without any help at 3 months after surgery, exhibiting an increase in the HHS to a mean value of 71.5 points (range 52–93 points), with no significant differences among groups regarding the preoperative Crowe score that had led to the operation.

In total, 71 (79.8%) stems were positioned in a neutral alignment, 11 (12.3%) in slight varus, and 7 (7.9%) in slight valgus. Radiographic evaluation demonstrated osteointegration of the implants with stable bone growth observed at the stem–endosteum interface; signs of bone readaptation and thinning of the femoral calcar were present in nine hips; and pedestal formation was never observed. There were no cases of subsidence exceeding 3 mm or evidence of impending component failure, no cases of implant dislocation, and no cases of component breakage. None of the patients underwent revision for septic or aseptic loosening of the stem and/or of the cup; none sustained a periprosthetic fracture. No early infection or wound healing problems occurred.

**Fig. 2:**
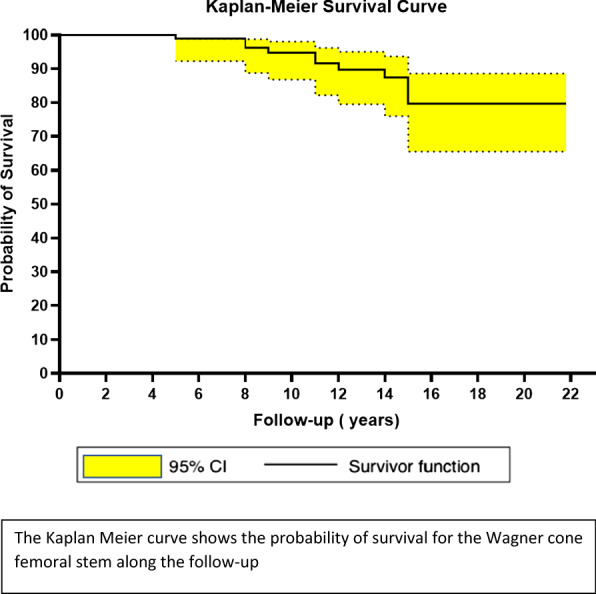
The Kaplan–Meier curve: survival rate of 99.2% at 5 years, 95.1% at 10 years, and 81.3% at 20 years

The Kaplan–Meier curve showed a survival rate of the Wagner cone stem of 99.2% (95% CI 92.8–100) at 5 years, 95.1% (95% CI 87.5–98.9) at 10 years, and 81.3% (95% CI 88.7–66.4) at 20 years (Fig. 2).

Heterotopic ossifications were found in seven (7.9%) cases, of which three cases were stage 1, three cases stage 2, and one case stage 3. No case was observed at stage 4. Ossifications were developed within the first year of follow-up, without any evident joint limitation and showing no changes over time in all cases. No patients required further surgery for ossification removal. One intraoperative complication was reported: one patient experienced an intraoperative fracture of the greater trochanter that was managed with braided cables and healed with limiting active abduction and dragging load on the operated limb for 6 weeks. One patient underwent a deep venous thrombosis on the fifth postoperative day that resolved without sequelae with medical therapy. Another patient, 14 years after her THA, had a reoperation for a suspected loose acetabular cup (Protek Fitek) due to radiographic evidence of sovra-acetabular osteolysis, which was bone ingrown and left in place; the lytic areas were filled with morcellized autologous bone graft, and a head and liner exchange was completed.

## Discussion

Coxarthrosis secondary to DDH leads to pathological bony anatomy and severe biomechanical alterations around the hip joint, increasing the difficulty of THA, which remains the treatment of choice for end-stage arthritis of the hip. In such an abnormal proximal femoral morphology, a stem with metaphyseal fitting flat-wedge taper or fit and fill should not be considered appropriate [9, 21], while a diaphyseal engaging stem that dials in the desired amount of anteversion is desirable, owing to its capability of adaptation in small femurs, with poor metaphyseal bone quality, and possibly with previous hip surgeries. Its use should therefore minimize the risk of threatening the long-term survival of the prosthetic implant related to aseptic loosening. Furthermore, with a conical prosthesis, initial fixation is secured by the longitudinal sharp ribs, providing a uniform axial load transfer, greater proximally than distally, which may prevent stress-shielding and promote proximal osteointegration of the stem.

In our series, radiological evaluation at follow-up showed osteointegration of the stem in the proximal region of the femur, even with signs of thinning of the femoral calcar in nine hips, and no pedestal formation distally, this phenomenon likely being due to the conical geometry of the implant.

Although poor outcome related to mechanical failure due to subsidence of the femoral component is a major concern when using a diaphyseal-fitting implant such as the Wagner cone, the revision rate for aseptic loosening of the stem at an average follow-up of 11.7 years is 0%. This outcome is in line with previously reported results with conical tapered stem: Faldini et al. reported that none of their 28 implants had to be revised at an average follow-up of 12 years [22]. In 52 THAs performed on patients with a small physique and younger than 40 years, with a mean duration of follow-up of 7.7 years, subsidence was present in three Wagner cones, but none of the implants were revised [14]. In another series of 173 implants with a mean follow-up of 87 months, revision was required in two cases owing to periprosthetic fracture [2]. Revision occurred in 2 of 102 complex hip replacements combined with femoral shortening osteotomy for Crowe IV DDH, with a survival of 95.9% at 10 years, in the study group of Grappiolo et al. [5]. According to Zhang, in 59 THAs performed on patients with small or abnormal proximal femoral anatomic proportions with a follow-up to 7 years, only one patient underwent revision surgery as a result of late infection, while no progressive radiolucencies were observed, and radiographic evaluation demonstrated stability of all implants [9]. It has been shown that there are fewer implant-related complications in patients undergoing THA with a dislocated hip classified as Crowe type IV when cylindrical stems 2/3 coated were used to reconstruct a step-cut osteotomized femur compared with tapered stems with 1/3 proximal coating [21].

We are well aware that coxarthrosis secondary to DDH is a diagnosis that theoretically may expose the implant to instability, due to the fact that a head with a diameter smaller than 32 mm biomechanically worsens the range of motion and the jump distance becomes shorter, leading to subluxation and/or dislocation. In osteoarthritis secondary to developmental dysplastic hip, a cup with a small diameter is often implanted, and the use of a femoral head with a diameter of 28 mm is a mandatory choice because the diameter of the femoral head is strictly related to the outer diameter of the cementless shell. Theoretically, prosthetic heads need to be enlarged to achieve better stability, which would imply that the liner thickness became thinner; a decrease in the polyethylene liner thickness or a decrease in the head-liner conformity leads to higher peak contact stresses, smaller contact areas, and, consequently, lower biomechanical wear factor [23, 24]. In this report, the use of a femoral head diameter of 28 mm was reserved for 56 of the 100 implants, mainly because the outer diameter of the cementless shell was 48 mm or less in 46 THAs. We have registered no cases of dislocation; in our opinion, it is likely related to three main factors that may protect from this complication: the choice of the direct lateral approach, the small numbers of hips that had had previous proximal femoral osteotomy, and, overall, the fact that none of the operations were performed on Crowe IV hips. In such high dislocated hips, modular stems may be used and often further surgeries are required, such as femoral metaphyseal subtrochanteric shortening osteotomy to balance the leg length and pelvic obliquity, and occasionally trochanteroplasty in an attempt to avoid impingement between the trochanter and iliac wing, thus leading to high risk of instability [1–6, 11, 15, 25–27].

There are some limitations to this study. Firstly, there is inherent bias in retrospective studies based on the design, with 11 implants of the initial 100 THAs lost at the final follow-up. Secondly, there is no control group with other femoral implants. Thirdly, it took a long time to collect 100 THAs, performed by six different surgeons, although using the same approach, likely because the number of patients enrolled in this study was limited by the number of surgeons involved in the operations. Finally, we have not measured important clinical and biomechanical parameters such as leg length discrepancy and femoral offset, even if their improvement can be postulated owing to all the implants having been stable at the latest follow-up.

In conclusion, this study confirms the theoretical advantages that suggest the choice of the Wagner cone and might support its application when technical difficulties during prosthetic surgery are expected because of abnormal proximal femoral anatomy.

**Table 1:** Implant features

Size of arthroplasty	Number
Diameter of cup (mm)	
58	2
56	6
54	7
52	11
50	28
48	20
46	12
44	14
Total	100
Size of stem (mm)	
24	3
23	2
22	6
21	4
20	7
19	10
18	13
17	14
16	26
15	11
14	4
Total	100
Diameter of head (mm)	
36	6
32	38
28	56
Total	100
Coupling bearing	
Ceramic-on-polyethylene	57
Metal-on-polyethylene	23
Ceramic-on-ceramic	20
Total	100

## Data Availability

The datasets generated and analyzed during the current study are not publicly available because the data are not public, but are available from the first author on reasonable request.

## Abbreviations

THA: Total hip arthroplasty
DDH: Developmental hip dysplasia
BMI: Body mass index
HHS: Harris Hip Score
